# Insulin Glargine U100 Utilization in Patients with Type 2 Diabetes in an Italian Real-World Setting: A Retrospective Study

**DOI:** 10.1155/2019/3174654

**Published:** 2019-12-31

**Authors:** Luca Degli Esposti, Valentina Perrone, Stefania Saragoni, Valerio Blini, Stefano Buda, Rosella D'avella, Gina Gasperini, Fabio Lena, Francesca Fanelli, Luca Gazzi, Francesco Giorgino

**Affiliations:** ^1^Clicon S.r.l. Health, Economics & Outcomes Research, Ravenna, Italy; ^2^Complex Operation Unit-Pharmaceutical Department of Arezzo-Toscana Sud Est Local Health Unit, Arezzo, Italy; ^3^Complex Operation Unit of Hospital Pharmacy for Hospital of Siena-Territory Continuity of Care, Toscana Sud Est Local Health Unit, Siena, Italy; ^4^Local Health Unit-Pharmaceutical Department of Grosseto, Toscana Sud Est Local Health Unit, Grosseto, Italy; ^5^Sanofi S.p.A, Italy; ^6^Department of Emergency and Organ Transplantation, Section of Internal Medicine, Endocrinology, Andrology and Metabolic Diseases, University of Bari Aldo Moro, Bari, Italy

## Abstract

**Background:**

This study is aimed at estimating the proportion of type 2 diabetes mellitus (T2DM) patients treated with basal insulin (insulin glargine U100) and at evaluating daily insulin dose, treatment pattern, and adherence to treatment of these patients.

**Methods:**

Data from administrative and laboratory databases of 3 Italian Local Health Units were retrospectively collected and analyzed. All patients with a diagnosis of T2DM between 01/01/2012 and 31/12/2012 were considered, and those with at least a prescription of insulin glargine between 01/01/2013 and 31/12/2014 were included and followed up for one year. For each patient, we evaluated HbA1c levels both at baseline and during the follow-up period and the daily average dose of insulin. Medication adherence was defined by using medication possession ratio (MPR) and reported as proportion of patients with MPR ≥ 80%.

**Results:**

7,422 T2DM patients were available for the study. According to the antidiabetic medication prescribed, patients were categorized into four groups: insulin glargine only, insulin glargine plus oral glucose-lowering drugs, insulin glargine plus rapid-acting insulin, and insulin glargine plus DPP-4 inhibitors. Median daily dose of insulin among insulin glargine only patients was higher than in other groups (35 IU vs. 20 IU, *p* < 0.05), and a higher percentage of them achieved a target HbA1c value of less than 7.0% (53.8% vs. 30%, *p* < 0.05), and a higher percentage of them achieved a target HbA1c value of less than 7.0% (53.8% vs. 30%, *p* < 0.05), and a higher percentage of them achieved a target HbA1c value of less than 7.0% (53.8% vs. 30%,

**Conclusions:**

A large proportion of T2DM patients treated with insulin fail in achieving the glycemic target of HbA1c level < 7%, irrespective of treatment regimen; however, basal insulin only is associated with lower therapeutic unsuccess. Adherence to antidiabetes medications is also suboptimal in these patients and should be addressed to improve long-term outcomes of reducing and preventing microvascular and macrovascular complications.

## 1. Introduction

Diabetes mellitus is a chronic common metabolic disorder [1]. In 2014, the global prevalence of diabetes was estimated to be 9% among adults aged more than 18 years [2]. In 2016, more than 3.2 million people in Italy were reported to suffer from diabetes, representing 5.3% of the total population (16.5% among people aged 65 and over) [3].

Recent epidemiological studies have shown an increasing prevalence of diabetes in Europe and worldwide [4–6]. Type 2 diabetes (T2DM) is the most common form of the disease, corresponding to approximately 90% of cases. From the onset of the disease until the symptoms develop, many people with undiagnosed diabetes may already have complications such as chronic kidney disease, heart failure, ischemic heart disease, retinopathy, and neuropathy [7–9].

The disease burden related to diabetes is thus high and rising in every country, supported by the global rise in the prevalence of obesity and unhealthy lifestyles [2, 10]. Indeed, diabetes and its complications are the major causes of death in most countries [7].

During the past few years, there have been significant advances in diabetes medications, insulin delivery systems, and glucose monitoring technologies. The latest evidence-based guidelines recommend that the appropriate therapeutic regimen for each patient with diabetes depends on the patient's characteristics and individual needs and circumstances [11–13].

Several treatment guidelines include modifications of adverse lifestyle factors, noninsulin oral antidiabetes therapy, and/or insulin therapy associated with self-monitoring of blood glucose to implement glycemic control [11–14].

Achieving long-term glycemic control is key for the reduction and prevention of microvascular and macrovascular complications [11–14]. Most guidelines consider a glycated hemoglobin A1c (HbA1c) value < 7% (53 mmol/mol) as the general target for glucose control, while more stringent targets, i.e., HbA1c < 6.5% (48 mmol/mol), should be pursued in specific patients [7]. Despite extensive evidence on the importance of control of blood glucose, glycemic targets are often not achieved [13].

Systematic reviews of observational studies have shown that the effectiveness of diabetes management largely relies upon patients' compliance with recommendations and treatment. Assessments of adherence rates to antidiabetes drugs showed variable results, but generally indicated a strong tendency toward poor medication adherence [15, 16]. A recent review by Krass et al. systematically evaluated a total of 27 studies on adherence to diabetes medications and reported levels of adherence ranging from 39% to 93%, with only a few studies showing an adherence level ≥ 80%, which is generally considered as the threshold for determining adherent patients [16, 17]. Identifying barriers to diabetes management is thus important to improve adherence in diabetes care in a real-world context.

The objectives of this observational database study, conducted in real-world setting, were (i) to estimate the prevalence of patients diagnosed with T2DM treated with basal insulin (insulin glargine U100) and describe their demographic and clinical characteristics, including comorbidities, cotreatments, and metabolic control; (ii) to evaluate the pharmacoutilization of insulin glargine alone or in combination therapies with other antihyperglycemic agents in patients diagnosed with T2DM treated with basal insulin in terms of daily insulin dose, treatment pattern, and adherence to treatment.

## 2. Methods

### 2.1. Data Sources

The study was based on the analysis of both administrative and laboratory databases of 3 Italian Local Health Units (LHUs), including approximately 850,000 health-assisted individuals.

To perform the analysis, the following databases were used: (i) beneficiaries database; (ii) pharmaceuticals database, which includes the Anatomical-Therapeutic-Chemical (ATC) code of the drug dispensed, the number of packs dispensed, the number of units per pack, the dose, the unit cost per pack, and the prescription date; (iii) hospitalization database, which includes all hospitalization data with the primary and secondary discharge diagnosis codes classified according to the International Classification of Diseases, Ninth Revision, Clinical Modification (ICD-9-CM); (iv) laboratory tests and specialist visits database; and (v) laboratory test value database.

The patient code in each database allowed electronic linkage between all different databases. To guarantee the patients' privacy, an anonymous univocal numeric code was assigned to each subject included in the study. No identifiers related to patients were provided to the researchers. All results were produced in an aggregate way. According to the Italian Guidelines regarding the conduction of observational studies [18], established by the Italian Drug Agency, “Agenzia Italiana del Farmaco—AIFA” on March 20, 2008, this study has been notified to the local Ethics Committee of each participating LHUs, and each participating LHU has approved the protocol. Informed consent was not obtained, since it is not required when using encrypted retrospective information for research purposes.

### 2.2. Study Population

An observational retrospective, cohort study was conducted. All patients with a diagnosis of T2DM between January 1, 2012 and December 31, 2012 (*enrolment period*) were included in the analysis. Patients were defined as having T2DM if, during the enrolment period, they had at least one hospitalization with a discharge diagnosis of T2DM (ICD-9-CM codes: 250.x0; 250.x2) or were aged ≥40 years and had at least one prescription of an antidiabetes agents (ATC code = A10B) with or without insulin (ATC code = A10A), respectively [19].

Only the cohort of patients who received at least one prescription of insulin glargine (ATC code = A10AE04) between January 1, 2013 and December 31, 2014 (*inclusion period*) was specifically considered for the analysis.

The index date was defined as the date of first prescription of insulin glargine during the inclusion period; starting from this date, individual patients were followed for one year, while the year before the inclusion date was used to characterize these patients (*follow-up* and *characterization periods*, respectively). Patients who were transferred to different LHU (outside the 3 included in this study) during the follow-up period were excluded from the analysis.

### 2.3. Study Variables

The following antihyperglycemic agents were evaluated: *insulin glargine* (ATC code: A10AE04); rapid-acting: insulin and analogues for injection, rapid-acting (human insulin ATC code: A10AB01), lispro insulin (ATC code: A10AB04); aspart insulin (ATC code: A10AB05); glulisine insulin (ATC code: A10AB06); *basal*: insulin and analogues for injection, intermediate-acting (human insulin ATC code: A10AC01), lispro insulin (ATC code: A10AC04), detemir insulin (ATC code: A10AE05), degludec insulin (ATC code: A10AE06); *mix*: insulin and analogues for injection, intermediate- or long-acting combined with fast acting (human insulin ATC code: A10AD01), lispro insulin (ATC code: A10AD04), aspart insulin (ATC code:A10AD05); *Glp-1*: analogues of glucagon like peptide-1 (ATC code = A10BJ, lixisenatide excluded); lixisenatide (ATC code: A10BJ03); DPP-4 inhibitors: inhibitors of dipeptidyl-peptidase IV (ATC code: A10BH) alone or in association with metformin (ATC codes: A10BD07; A10BD08; A10BD13); *other oral antidiabetic drugs*: biguanides (ATC code: A10BA); sulfonylureas (ATC code = A10BB); combination of oral blood glucose-lowering drugs (ATC code: A10BD, combination with DPP-4 excluded; alpha-glucosidase inhibitors (ATC code: A10BF); thiazolidinediones (ATC code: A10BG); *other blood glucose-lowering drugs* (ATC code: A10BX) excluding insulins; SGLT2: sodium-glucose cotransporter 2 inhibitors (ATC code: A10BK).

Data on baseline characteristics, including demographics (age and gender) and medical history (hospital admission, procedure, prescribed drugs, and profile of comorbidity), were collected during the year before the index date (*characterization period*). Previous use of antihypertensive [at least 2 prescriptions of antihypertensive medications (ATC code: C02, C03, C07-9)]; lipid-lowering agents [at least 2 prescriptions of medications for dyslipidemia (ATC code: C10)]; anti-inflammatory drugs [at least 2 prescriptions of anti-inflammatory drugs (ATC code: M10)]; and respiratory disease agents [at least 2 prescriptions of medications for chronic obstructive pulmonary disease (ATC code: R03)] were also evaluated. The following events and conditions were also examined: previous cardiovascular hospitalizations [at least 1 hospitalization with a primary or secondary ICD-9-CM code diagnosis of hypertensive disease (ICD-9 codes: 401-405)], acute myocardial infarction (ICD-9 code: 410), coronary disease (ICD-9 codes: 411–414), heart failure (ICD-9 code: 428), stroke and other cerebral circulatory dysfunction (ICD-9 codes: 430-438); arteriosclerosis of the main arteries and aneurysm (ICD-9 codes: 440-442); renal function [at least one hospitalization discharge diagnosis of chronic glomerulonephritis (ICD-9 code: 582), nephritis and nephropathy not specified as acute or chronic (ICD-9 code: 583), chronic kidney disease (ICD-9 code: 585), renal failure, unspecified (ICD-9 code: 586) during the characterization period]. Hypoglycemia was evaluated as a value of glycemia < 50 mg/dl in the last measurement prior to the index date. The use of a glycemic laboratory value instead of a hypoglycemic discharge diagnosis code lets to avoid an underestimation of hypoglycemia cases, because previous studies have shown that only one-third of the overall cases of hypoglycemic episodes occurring in subject with diabetes resulted in hospital admission and, of these, only few cases had a hypoglycemia diagnosis code [20]. Comorbidities were measured using the Charlson Comorbidity Index (CCI) [21] that assigns a score to each concomitant disease identified through treatments and hospitalizations during the characterization period; the CCI score reflects a patient's overall health status. This same methodology has been widely used as a way to compare disease severity in observational retrospective studies when data are unavailable [22, 23].

Among the included patients, the HbA1c level was evaluated both at baseline (index date) and during the follow-up period, respectively. The HbA1c levels were stratified as <7, ≥7% < 8%, ≥8% < 9%, and ≥9% [24, 25]. Daily doses of insulin for treatment groups, according to insulin regimen during the follow-up period, were also assessed.

Naïve patients were defined as those who had no prior insulin glargine prescription filled during the year preceding the index date.

### 2.4. Medication Adherence

The daily average dose was calculated by dividing the dose between the first and the penultimate prescription for the number of days between the index and the last prescription dispensed during the 12 months of the follow-up period. For each patient, the duration of treatment was calculated by dividing the total prescribed dose divided by the average daily dose. The medication adherence to insulin glargine during the follow-up period was measured by the calculating the MPR, defined as the percentage of time a patient had access to the medication, i.e., the duration of treatment divided by the duration of the follow-up period (365 days or less in case of death). Adherence was reported as proportion of patients with MPR > 80% [26].

### 2.5. Statistical Analysis

Continuous variables are reported as the mean value and standard deviation (SD) or median and interquartile range; categorical variables are shown as percentages and absolute numbers. To test differences between groups, we used Pearson's *χ*^2^ test for categorical data; in case of continuous variables, we used the analysis of variance (ANOVA) to test hypotheses about the means and Kruskal-Wallis to test hypotheses about medians. Differences were considered statistically significant with *p* < 0.05. All analyses were performed using STATA SE, version 12.0, and data management was carried out using Microsoft SQL Server 2012.

### 2.6. Study Endpoints

1. Description of demographic and clinical characteristics of T2DM patients treated with insulin glargine U100
2. Pharmacoutilization of insulin glargine in T2DM patients (evaluation of daily dose, treatment pattern, and adherence to treatment)

## 3. Results

**Figure 1:**
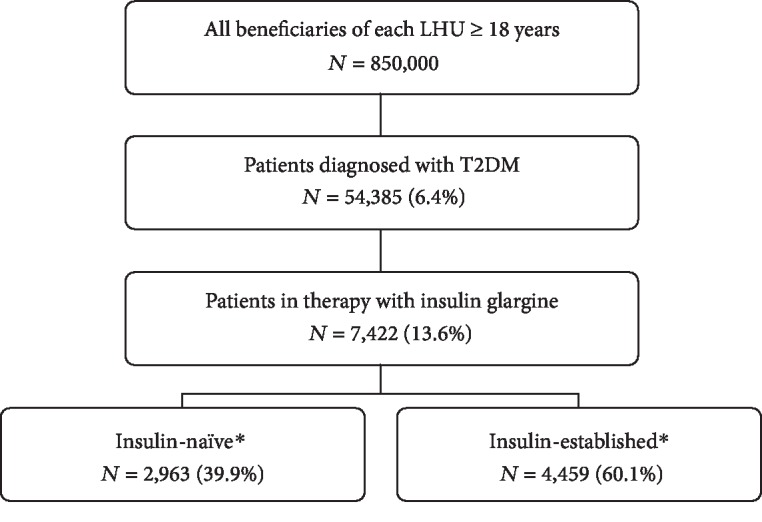
Flow chart of included patients. N: number; LHU: Local Health Unit; T2DM: diabetes mellitus type 2. ^∗^Insulin-naïve and insulin-established are referred to the absence or presence of therapy with insulin glargine, respectively, during the characterization period (12 months before the index date).

During the study enrolment period (2012), we identified 54,385 patients with a diagnosis of T2DM (about 6.4% of the health-assisted individuals).  Figure 1 shows the details of the inclusion criteria. Of the 54,385 patients identified in the database, 7,422 were on treatment with insulin glargine between January 1, 2013 and December 31, 2014, and the analyses were conducted on these subjects.

Among insulin glargine users, 2,963 were defined as insulin-naïve, and 4,459 were defined as insulin-established at baseline. Insulin-naïve are defined as those patients without prescriptions of insulin glargine in the period before the index date.

### 3.1. Treatment Patterns Based on Insulin Glargine

The combination between insulin glargine and other antihyperglycemic agents was evaluated during the 6 months after the index date (Table 1).

### 3.2. Demographic and Clinical Features of Insulin Glargine T2DB Patients

The baseline demographic and clinical characteristics of the included patients, stratified according to the treatment assigned during the 6 months after the index date, are described in Table 2. Gender was almost equally distributed among users of insulin glargine only, insulin glargine plus other oral glucose-lowering drugs, and insulin glargine plus rapid-acting insulin (on overage, males: 47.6%, 51.1%, and 51.1%, respectively); the percentage of males among patients initiating insulin glargine plus DPP-4 inhibitors resulted 55.5%. Among patients initiating insulin glargine only, insulin glargine plus other oral glucose-lowering drugs, and insulin glargine plus rapid-acting insulin, the mean age (±SD) was found to be 71.7 (±14.6), 71.8 (±11.9), and 69.3 (±13.3) years, respectively. Patients initiating insulin glargine plus DPP-4 inhibitors were younger than other patients' groups (67.7 ± 10.0 years), *p* < 0.05.

History of hypertension and dyslipidemia was higher in patients on insulin glargine plus DPP-4 inhibitors than in other patients included (for hypertension: 77.0% vs. 65.0% in those on insulin glargine only, 75.6% in those on insulin glargine plus other oral glucose-lowering drugs, 73.5% in those on insulin glargine plus rapid-acting insulin; for dyslipidemia: 60.2% vs. 32.8% in those on insulin glargine only, 50.6% in those on insulin glargine plus other oral glucose-lowering drugs, 51.2% in those on insulin glargine plus rapid-acting insulin). At baseline, the percentage of hypoglycemia was higher among insulin glargine plus rapid-acting insulin and insulin glargine plus DPP-4 inhibitors users than in the other treatment groups (1.3% and 1.4%, respectively, vs. 0.6% insulin glargine only and 0.7% insulin glargine plus other oral glucose-lowering drugs), but the results were not statistically different. Patients on treatment with insulin glargine only showed a higher percentage of previous cardiovascular (CV) events than the other treatment groups (28.3% vs. 14.6% insulin glargine plus other oral glucose-lowering drugs, 19.1% insulin glargine plus rapid-acting insulin, 10.9% insulin glargine plus DPP-4 inhibitors).

### 3.3. Relationship between Daily Insulin Dose and HbA1c Levels

The correlation between the daily dose (IU, mean, median) of insulin glargine and the level of HbA1c stratified according to the therapeutic choice is shown in Table 3. The analysis was performed for different levels of HbA1c (i.e., <7.0%, ≥7.0% < 8.0%, ≥8.0% < 9.0%, ≥9.0%) and different levels of daily median insulin dose (i.e., <20 IU, 20-29 IU, 30-39 IU, ≥40 IU). The analysis was carried out only among patients with available HbA1c levels, and percentages were calculated for each daily median dose. Among insulin glargine only users, the mean (median) daily dose was 42.4 IU (35 IU); in this subgroup, 53.8% of patients achieved an HbA1c value of less than 7.0%. Median daily doses of insulin for the other treatment groups (insulin plus other oral glucose-lowering drugs, insulin glargine plus DPP-4 inhibitors, insulin glargine plus rapid-acting insulin) were lower (approximately 20 UI), *p* < 0.05, and only 30% of patients achieved an HbA1c value < 7.0%.

Table 4 shows the correlation between the daily mean insulin dose and the last value of HbA1c during the observation period in the insulin glargine only cohort. A higher insulin dose was generally associated with a higher percentage of patients who reached the glycemic target of HbA1c < 7.0 during follow-up, but the results were not statistically different.

### 3.4. Adherence to Treatment

**Figure 2:**
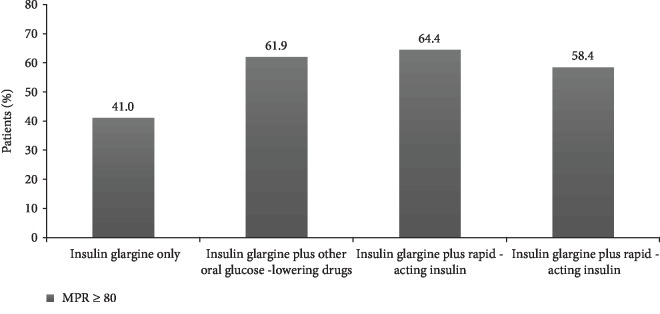
Percentage of adherent patients (MPR ≥ 80%) stratified according to therapeutic regimen.

According to the defined threshold of MPR ≥ 80%, 41% of the insulin glargine only patients were considered adherent, while the percentage of adherent patients was 61.9%, 64.4%, and 58.4% for insulin plus other oral glucose-lowering drugs, insulin glargine plus DPP-4 inhibitors, and insulin glargine plus rapid-acting insulin cohorts, respectively (Figure 2), *p* < 0.001.

## 4. Discussion

In clinical practice, optimal glycemic control is difficult to obtain on a long-term basis due to multiple potential factors influencing the outcome, as age, sex, education, smoking, clinical characteristics of the patient, and type of medication [27]. Nevertheless, achievement of glycemic control remains the major therapeutic objective for prevention of target organ damage and other complications arising from diabetes. Intervention goals should be tailored to the individual patient and should take into account the patient's preferences, presence of diabetic complications, and presence of comorbidity and life expectancy.

The landscape of glucose-lowering medications for T2DM has changed dramatically over the past two decades [28–33]. In this context, the treatments available have grown in complexity, with older classes being replaced by increasing utilization of newer glucose-lowering agents, such as DPP-4 inhibitors and newer insulin analogs and their formulations. [29, 30] However, in the past few years, glycemic control of T2DM has not substantially improved in the overall population with a significant proportion of diabetic patients still experiencing inadequate control.

The data from our retrospective analysis add to the growing body of evidence showing that a significant proportion of patients treated with insulin are failing to achieve glycemic targets in the real world, indicating the need to increase the daily dose. Indeed, an intensification approach should be considered for patients who receive large doses of basal insulin without reaching fasting blood glucose and glycated hemoglobin target levels [27]. Our study provides clinical information on some outcomes associated with each type of insulin-intensification strategies, based on real-life everyday clinical practice.

Adherence to antidiabetes medications is crucial to reach metabolic control, since nonadherence is associated with increased levels of HbA1c as well as other negative outcomes such as increased LDL cholesterol levels, frequent hospitalizations, and mortality [34]. Consistent with previous studies [16, 26], we found that adherence to antihyperglycemic medications was suboptimal among patients with diabetes on insulin therapy. Scientific evidence suggests that patients with poor adherence to prescribed antidiabetes medications have significantly high prevalence of poor glycemic control when compared with those with high and medium medication adherence. Furthermore, recent registration trials and postmarketing studies have suggested that newer long-acting insulin analogs may address some of the unmet needs of current basal insulin options in terms of risk of hypoglycemia, dosing time inflexibility, treatment adherence, and treatment satisfaction [35].

The health-related costs associated with T2DM morbidity and mortality are continually increasing and are exacerbated by resulting long-term complications [36]. Significant evidence exists showing the relationship between adherence to antidiabetic medications and health care costs in adults with T2DM [37]. Generally, nonadherence to T2DM medications not only results in poor health outcomes but it also has a significant impact on healthcare costs [37]. A recent review by Kennedy-Martin et al. highlighted that, although patients who were adherent had higher drug costs, their overall diabetes-related medical costs (consisting of hospitalization, ambulatory, inpatient, emergency room visits, and other costs) were lower [37]. Additional studies have found similar results, reporting that overall medical cost savings outweigh increased costs from more frequent prescription drug use.

We acknowledge some limitations of our study. Our cohort of patients reflected real clinical practice, and the results must be interpreted, taking into account of limitations related to the observational nature of the study, based on data collected through administrative and laboratory databases. Administrative data are not intently gathered for research purpose, nevertheless they represent remarkable resources for healthcare research, by providing a large and representative sample of study population without requiring the personal involvement of participants. [38] The main limitations of using these data sources concern (i) different coding criteria [39], which was not an issue in this study, as we were able to join different databases thanks to univocal patient code and (ii) missing data due to inaccurate or lost medical records. We do recognize the latter as one of the limitations of the present study as well as the lack of clinical information, such as data on comorbidities, the severity of the pathology, insulin dosage adjustments for body weight, and other potential confounders that could have influenced our results. The results and conclusions of this study are limited to the population analyzed.

## 5. Conclusions

The results of this study, based on data from clinical practice, show that a large proportion of T2DM patients treated with insulin fall to achieve glycemic targets, irrespective of the specific treatment regimen. However, use of basal insulin only is associated with lower therapeutic unsuccess. On the other hand, adherence to antidiabetes medications appears to be also suboptimal in these patients and should be addressed to improve long-term outcomes.

## Figures and Tables

**Table 1:** Different combinations of insulin glargine and other hypoglycemic therapies evaluated 6 months after the index date (first prescription of insulin glargine).

Cohort of study	Total		Insulin-naïve		Insulin-established		*p* value^∗^
	*N*	%	*N*	%	*N*	%	
Insulin glargine only	466	6.3	293	9.9	173	3.9	<0.001
Insulin glargine+other oral glucose-lowering drugs	1,590	21.4	680	22.9	910	20.4	0.002
Insulin glargine+rapid-acting insulin	4,475	60.3	1,576	53.2	2,899	65.0	<0.001
Insulin glargine+DPP-4 inhibitors	357	4.8	167	5.6	190	4.3	0.004
Insulin glargine+other combinations	534	7.2	247	8.3	287	6.4	0.002
Total	7,422	100.0	2,963	100.0	4,459	100.0	

∗Insulin-naïve vs. insulin-established.

**Table 2:** Demographic and baseline clinical characteristics stratified according to insulin-based therapeutic regimens.

	Insulin glargine only (*n* = 466)			Insulin glargine plus other oral glucose-lowering drugs (*N* = 1,590)			Insulin glargine plus rapid-acting insulin (*N* = 4,475)			Insulin glargine plus DPP-4 inhibitors (*N* = 357)		
	Total	Insulin-naïve	Insulin-established	Total	Insulin-naïve	Insulin-established	Total	Insulin-naïve	Insulin-established	Total	Insulin-naïve	Insulin-established
*N* (%)	466	293 (62.9)	173 (37.1)	1,590	680 (42.8)	910 (57.2)	4,475	1,576 (35.2)	2,899 (64.8)	357	167 (46.8)	190 (53.2)
Age (mean ± SD)	71.7 ± 14.6	71.8 ± 14.9	71.6 ± 14.2	71.8 ± 11.9	71.6 ± 12.6	71.9 ± 11.4	69.3 ± 13.3	69.5 ± 14.0	69.1 ± 12.9	67.7 ± 10.0^^^	67.4 ± 11.2°	68.0 ± 8.9^§^
Male (%)	47.6	45.4	51.4	51.1	49.6	52.2	51.1	51.1	51.1	55.5	54.5	56.3
Hypertension (%)	65.0	62.1	69.9	75.6	72.6	77.8	73.5	70.2	75.3	77.0^^^	70.7°	82.6^§^
Dyslipidemia (%)	32.8	29.7	38.2	50.6	43.5	55.8	51.2	40.0	57.3	60.2^^^	52.7°	66.8^§^
Anti-inflammatory agents (%)	14.4	14.3	14.5	18.1	17.8	18.2	15.8	15.0	16.2	17.4	15.6	18.9
Asthma/COPD (%)	10.3	9.9	11.0	9.7	11.6	8.4	10.1	10.3	10.0	6.2	5.4	6.8
Kidney disease	8.6	8.5	8.7	3.4	4.1	2.9	6.3	8.2	5.3	3.4^^^	3.6°	3.2^§^
Hypoglycemia (%)	0.6	0.7	0.6	0.7	0.6	0.8	1.3	1.1	1.3	1.4	—	2.6
Previous CV events (%)	28.3	30.0	25.4	14.6	19.0	11.3	19.1	25.1	15.8	10.9^^^	9.6°	12.1^§^
Hypertensive disease (%)	14.4	16.0	12.5	9.2	13.9	7.2	10.4	11.6	6.8	7.0^^^	8.5°	6.8
Acute myocardial infarction (%)	2.4	3.1	1.3	1.1	2.3	0.6	1.8	1.2	1.0	0.8	1.6	1.1
Coronary disease (%)	8.2	7.5	5.3	4.0	9.3	5.4	7.6	9.2	3.0	6.4^^^	6.6°	7.4^§^
Heart failure (%)	7.7	8.9	5.0	3.5	8.0	3.0	5.8	5.8	2.4	2.2^^^	4.6°	1.6^§^
Stroke and other cerebral circulatory dysfunction (%)	11.4	10.9	6.2	5.0	7.4	1.2	5.3	12.1	4.2	1.7^^^	4.2°	2.1^§^
Arteriosclerosis of the main arteries and aneurysm (%)	1.7	2.4	2.1	1.8	2.1	0.6	2.4	0.6	1.5	1.4	2.6	2.1
HbA1c (%)^∗^												
<7%	26.5	24.4	30.9	18.4	15.9	20.6	16.8	16.7	16.9	15.5^^^	7.4^∗^	23.2^§^
≥7% < 8%	13.4	12.4	15.5	23.6	20.5	26.3	25.3	19.5	29.3	27.7	19.9	35.2
≥8% < 9%	8.8	9.1	8.2	16.7	15.3	18.0	19.1	16.5	20.8	19.4	22.1	16.9
≥9%	14.4	13.4	16.5	21.5	28.7	14.9	19.2	24.5	15.5	23.0	34.6	12.0
Not detected	36.9	40.7	28.9	19.9	19.6	20.1	19.6	22.7	17.5	14.4	16.2	12.7

^∗^Analysis carried out in the cohort of patients with available laboratory data. ^^^*p* < 0.05 among the insulin groups. °*p* < 0.05 among the insulin-naïve groups. ^§^*p* < 0.05 among the insulin-established groups.

**Table 3:** Correlation between daily insulin dose (IU, median) and HbA1c levels stratified according to the therapeutic regimen.

HbA1c	Insulin glargine only (*n* = 158)			Insulin glargine plus other oral glucose-lowering drugs (*n* = 901)			Insulin glargine plus DPP-4 inhibitors (*n* = 249)			Insulin glargine plus rapid-acting insulin (*n* = 2,097)		
	Patients with HbA1c value (%)	Daily dose (IU)		Patients with HbA1c value (%)	Daily dose (IU)		Patients with HbA1c value (%)	Daily dose (IU)		Patients with HbA1c value (%)	Daily dose (IU)	
		Mean (SD)	Median (IQR)		Mean (SD)	Median (IQR)		Mean (SD)	Median (IQR)		Mean (SD)	Median (IQR)
<7%	53.8	42.4 (35.1)	35.7 (37.5)	29.3%	34.1 (40.1)	20.9 (38.5)	34.1%	39.9 (32.4)	43.0 (37.2)	28.8^^^	40.4 (34.2)	20.9 (35.8)°
7-8%	19.6	32.6 (33.9)	15.3 (42.7)	35.2%	30.3 (39.3)	16.6 (29.2)	34.5%	34.0 (33.6)	19.7 (39.8)	35.1^^^	30.3 (46.7)	19.9 (24.5)
8-9%	13.9	34.6 (34.3)	18.7 (38.4)	19.1%	26.5 (32.7)	16.7 (22.2)	21.7%	30.6 (28.3)	17.1 (38.9)	22.2^^^	30.0 (34.4)	20.4 (23.8)
>9%	12.7	35.6 (27.7)	28.5 (36.9)	16.4%	30.7 (34.4)	18.2 (32.5)	9.6%	28.0 (26.0)	19.2 (27.8)	14.0^^^	29.5 (23.0)	22.7 (24.3)

The analysis was carried out on the cohort of patients for which the laboratory data were available and with at least 3 months of follow-up. ^^^*p* < 0.05 among groups. °*p* < 0.05 among groups.

**Table 4:** Daily mean insulin dose according to HbA1c levels in the insulin glargine only cohort (*N* = 158^∗^).

		Daily mean insulin dose (IU)				Total
		<20 IU	20 IU-29 IU	30 IU-39 IU	≥40 IU	
Last HbA1c during follow-up (%)	<7.0%	46.7	33.3	58.3	63.1	53.8
	≥7.0% < 8.0%	25.3	—	16.7	15.4	19.6
	≥8.0% < 9.0%	17.3	33.3	8.3	9.2	13.9
	≥9.0%	10.7	33.3	16.7	12.3	12.7

^∗^The analysis was carried out on the cohort of patients for which laboratory data were available and with at least 3 months of follow-up. The last value of HbA1c during the follow-up period was considered. *p* = 0.256 among different levels of HbA1c and daily mean insulin dose.

## Data Availability

Data are not available due to ethical issues.
